# Statement of Retraction: The urinary exosomes derived from premature infants attenuate cisplatin-induced acute kidney injury in mice via microRNA-30a-5p/ mitogen-activated protein kinase 8 (MAPK8)

**DOI:** 10.1080/21655979.2024.2299611

**Published:** 2024-02-20

We, the Editors and Publisher of the journal *Bioengineered*, have retracted the following article:

Mingming Ma, Qiao Luo, Lijing Fan, Weilong Li, Qiang Li, Yu Meng, Chen Yun, Hongwei Wu, Yongping Lu, Shuang Cui, Fanna Liu, Bo Hu, Baozhang Guan, Huanhuan Liu, Shengling Huang, Wenxue Liang, Stanislao Morgera, Bernhard Krämer, Shaodong Luan, Lianghong Yin & Berthold Hocher (2022) The urinary exosomes derived from premature infants attenuate cisplatin-induced acute kidney injury in mice via microRNA-30a-5p/ mitogen-activated protein kinase 8 (MAPK8), Bioengineered, 13:1, 1650-1665, DOI: 10.1080/21655979.2021.2021686

Since publication, significant concerns have been raised about the integrity of the data and reported results in the article. When approached for an explanation, the authors did not provide their original data or any necessary supporting information. As verifying the validity of published work is core to the integrity of the scholarly record, we are therefore retracting the article. All authors listed in this publication have been informed.

We have been informed in our decision-making by our editorial policies and the COPE guidelines.

The retracted article will remain online to maintain the scholarly record, but it will be digitally watermarked on each page as ‘Retracted’.

